# 555 The Influence of Hypertension Management on Edema and Wound Outcomes in Lower Extremity Burn Patients

**DOI:** 10.1093/jbcr/iraf019.184

**Published:** 2025-04-01

**Authors:** Danielle Miller, Sheza Habib, Juquan Song, Amina El Ayadi

**Affiliations:** University of Texas Medical Branch; University of Texas Medical Branch; University of Texas Medical Branch; University of Texas Medical Branch

## Abstract

**Introduction:**

Burn patients with co-morbid hypertension are at elevated risk of complications, including edema and wound development. Hypertension may exacerbate burn recovery by increasing vascular resistance and systemic inflammation, which impairs tissue perfusion and delays wound healing. Edema, often exacerbated by hypertension, impairs local circulation and lymphatic drainage, further complicating recovery by promoting fluid accumulation and tissue damage. This study evaluates the relationship between hypertension, edema, and wound development in lower extremity burn patients.

**Methods:**

A retrospective analysis using the TriNetX database identified adult (>18 years) lower extremity (LE) burn patients. Patients were categorized into three group cohorts: (1) LE burns without a history of hypertension or hypertension treatment, (2) LE burns with a history of untreated hypertension, and (3) LE burns with hypertension and a history of treatment with antihypertensives (beta blockers, peripheral vasodilators, diuretics, RAAS agents, calcium channel blockers). The cohorts were balanced using propensity score matching of age at index, race, and ethnicity covariates. The incidence of lower extremity swelling or edema, and lower extremity wound development three months post-burn, to account for initial healing time, was assessed. Statistical analysis compared percent risk and number of instances, using t-tests with a significance threshold of p < 0.05.

**Results:**

Compared to Cohort 1, Cohort 2 had significantly more edema (p < 0.05), higher percent risk (5.7% vs 3.1%, p < 0.0001) of edema, and higher percent risk of lower extremity wounds (4.9% vs 2.3%, p < 0.0001). Cohort 3 had significantly higher percent risk of lower extremity edema (14.2% vs 1.7%, p < 0.0001) and superficial injuries or open wounds (11.9% vs 2.7%, p < 0.0001), compared to Cohort 1. Additionally, Cohort 3 had a higher risk of developing worse complications compared to Cohort 2, including increased superficial injuries or open wounds (11.6% vs 4.9%, < 0.0001) and edema (14.3% vs 4.1%, < 0.0001).

**Conclusions:**

The data suggests a correlation between preexisting hypertension and increased edema and wound complications in lower extremity burn patients. According to our data, hypertension management showed worse lower extremity burn complications. Further research is needed to clarify the role of specific antihypertensives on wound healing and to explore how hypertension severity impacts lower extremity burn outcomes.

**Applicability of Research to Practice:**

The findings suggest hypertension management and close monitoring with tailored cardiovascular interventions can reduce complications. This could influence clinical practice by encouraging early intervention in burn units, targeting patients with cardiovascular co-morbidities to improve recovery and reduce long-term complications.

**Funding for the Study:**

N/A

